# The combined analysis of urine and blood metabolomics profiles provides an accurate prediction of the training and competitive status of Chinese professional swimmers

**DOI:** 10.3389/fphys.2023.1197224

**Published:** 2023-06-14

**Authors:** Ruoyu Yang, Yi Wang, Chunhua Yuan, Xunzhang Shen, Ming Cai, Liyan Wang, Jingyun Hu, Haihan Song, Hongbiao Wang, Lei Zhang

**Affiliations:** ^1^ College of Rehabilitation Sciences, Shanghai University of Medicine and Health Sciences, Shanghai, China; ^2^ Surgery Ward, Shanghai Health Rehabilitation Hospital, Shanghai, China; ^3^ Shanghai Research Institute of Sports Science (Shanghai Anti-Doping Center), Shanghai, China; ^4^ Central Lab, Shanghai Key Laboratory of Pathogenic Fungi Medical Testing, Shanghai Pudong New Area People’s Hospital, Shanghai, China; ^5^ Department of Physical Education, Shanghai University of Medicine and Health Sciences, Shanghai, China; ^6^ Department of Pediatrics, Shanghai Pudong New Area People’s Hospital, Shanghai, China

**Keywords:** metabolomics, urine metabolites, swimmers, athletic status, identification model, nuclear magnetic resonance

## Abstract

**Objective:** The purpose of this study was to employ metabolomics for the analysis of urine metabolites in swimmers, with the aim of establishing models for assessing their athletic status and competitive potential. Furthermore, the study sought to compare the identification efficacy of multi-component (urine and blood) model versus single-component (urine or blood) models, in order to determine the optimal approach for evaluating training and competitive status.

**Methods:** A total of 187 Chinese professional swimmers, comprising 103 elite and 84 sub-elite level athletes, were selected as subjects for this study. Urine samples were obtained from each participant and subjected to nuclear magnetic resonance (NMR) metabolomics analysis. Significant urine metabolites were screened through multivariable logistic regression analysis, and an identification model was established. Based on the previously established model of blood metabolites, this study compared the discriminative and predictive performance of three models: either urine or blood metabolites model and urine + blood metabolites model.

**Results:** Among 39 urine metabolites, 10 were found to be significantly associated with the athletic status of swimmers (*p* < 0.05). Of these, levels of 2-KC, cis-aconitate, formate, and LAC were higher in elite swimmers compared to sub-elite athletes, while levels of 3-HIV, creatinine, 3-HIB, hippurate, pseudouridine, and trigonelline were lower in elite swimmers. Notably, 2-KC and 3-HIB exhibited the most substantial differences. An identification model was developed to estimate physical performance and athletic level of swimmers while adjusting for different covariates and including 2-KC and 3-HIB. The urine metabolites model showed an area under the curve (AUC) of 0.852 (95% CI: 0.793–0.912) for discrimination. Among the three identification models tested, the combination of urine and blood metabolites showed the highest performance than either urine or blood metabolites, with an AUC of 0.925 (95% CI: 0.888–0.963).

**Conclusion:** The two urine metabolites, 2-KC and 3-HIV, can serve as significant urine metabolic markers to establish a discrimination model for identifying the athletic status and competitive potential of Chinese elite swimmers. Combining two screened urine metabolites with four metabolites reported exhibiting significant differences in blood resulted in improved predictive performance compared to using urine metabolites alone. These findings indicate that combining blood and urine metabolites has a greater potential for identifying and predicting the athletic status and competitive potential of Chinese professional swimmers.

## 1 Introduction

As a competitive sport, swimming is characterized by its cyclical nature, with elite athletes demonstrating an extended developmental trajectory thus emphasizing the need for rigorous selection and training criteria that ensure optimal performance and competitive success (Lahart and Metsios, 2018). Following elite swimmers’ training, physiological parameters such as hemoglobin, hematocrit, creatine kinase, urea, lactic acid, testosterone, and cortisol levels in the blood are reliable indicators to assess athletes’ physical fitness (Julian et al., 2017; Pla et al., 2019; González-Ravé et al., 2023). Another approach to evaluate athletes’ physical fitness is through analyzing the metabolites such as urea, creatinine, ketone bodies, phosphate, and nitrogenous compounds in urine (Moreira et al., 2018). In an experimental study involving rats, both plasma and skeletal muscle specimens were procured from the subjects post-swimming. The findings revealed a significantly lower incidence of oxidative damage in those rats which had been supplemented with hydrolyzed protein prior to swimming. This reduction in oxidative damage might be attributed to elevated levels of plasma leucine, isoleucine, and methionine observed in these subjects (Wang et al., 2014). In a separate experimental study, swimming athletes were administered branched chain amino acids as a supplement prior to their swimming activity. In comparison to the control group, the athletes in the experimental group exhibited a reduced degree of muscle protein proteolysis. This observed phenomenon could potentially be associated with the minor alterations in urinary concentrations of urea nitrogen, hydroxyproline, and methyl histidine (Tang, 2006). In a regime of high-intensity intermittent swimming training, the supplementation of branched-chain amino acids, along with arginine and citrulline prior to swimming, appeared to enhance the competitive performance levels of the swimmers (Hsueh et al., 2018). The foregoing research findings suggest a certain correlation between alterations in physical performance and metabolic changes experienced during swimming. The exploration of critical metabolic indicators and their respective changes could potentially serve as an efficacious approach to furthering our understanding of the physiological responses during swimming.

Metabolomics, a high-throughput analytical approach, has been employed across various fields, including microbiology, human disease, motion analysis, as well as animal and plant research (Abdelhafez et al., 2020; Klein et al., 2021; Bauermeister et al., 2022; Deutsch et al., 2022). Untargeted metabolomics analysis techniques primarily utilize nuclear magnetic resonance (NMR) spectroscopy and chromatography-mass spectrometry (Heaney et al., 2019). NMR spectroscopy enables the detection and quantification of small molecules in large populations, revealing significantly altered micrometabolites that are challenging to detect using conventional methods (Castagné et al., 2017). This technique plays a pivotal role in identifying and quantifying small molecules in blood, urine, and other samples, enabling in-depth exploration beyond basic physiological indicators. In metabolomic analyses, researchers may employ single-sample blood or urine metabolomic analysis (Brezmes et al., 2022; Talmor-Barkan et al., 2022) or combined blood and urine metabolomics analysis (Pal et al., 2022). As a vital complementary method to genomics, transcriptomics, and proteomics, metabolomics has garnered increasing attention in exercise research (Muthubharathi et al., 2021). Frequently, blood, urine, or saliva samples are used for analysis in exercise metabolomics studies (Lv et al., 2022). The identification of biomarkers and prediction of athletes’ competitive level and physiological state through exercise metabolomics are crucial for screening and tailoring training programs for athletes at different levels (Khoramipour et al., 2022; Weiss et al., 2022). Metabolomics, the comprehensive study of small molecules or metabolites within biological systems, has found increasing application in the field of sports science (Khoramipour et al., 2022). By analyzing the biochemical changes in an athlete’s body, metabolomics can provide valuable insights into their physiological adaptations, training responses, and overall performance (San-Millán, 2019).

The assessment of specific sports skills is critical for athlete development as it not only distinguishes levels of performance but also predicts future development potential (Koopmann et al., 2020). The emergence of metabolomics, due to its high-throughput and non-target properties, can detect multiple metabolites at once, providing a powerful tool for detecting differences in physical fitness and performance among athletes (Wang et al., 2020; da Cruz et al., 2022). Metabolomics is an omics field that primarily focuses on the study of motor metabolism in biological fluids such as blood and urine (Khoramipour et al., 2022). By analyzing exercise metabolomics, it may provide some reference for developing training plans, dietary regimens, and preventive measures for sports injuries based on metabolic characteristics (Bongiovanni et al., 2019). A blood metabolomics analysis was performed on athletes who had undergone high-intensity exercise and had varying levels of output per stroke and blood pressure. Similarly, to gain insight into the relationship between players’ movement patterns, metabolic characteristics, and different positions, saliva samples from elite male basketball players were collected and subjected to metabolic analysis. The findings showed that backcourt players had more aerobic metabolites, while frontcourt players exhibited more anaerobic metabolites (Khoramipour et al., 2022). Furthermore, metabolic profiling of athletes’ blood after the ingestion of nutritional β-glucan was conducted to determine whether it had an impact on their muscle strength. After supplementing with β-glucan for 4 weeks, the mean grip strength, right hand grip strength, left triceps strength, and upper limb muscle mass of athletes significantly increased, and there was a statistically significant difference in mean grip strength and right-hand grip strength compared to the control group. The increase in athlete muscle strength may be related to β-glucan affecting creatine-related pathways to increase aerobic endurance and enhance immune function. This measure is simple and has a direct correlation (Wang et al., 2022). Several researchers have successfully differentiated professional athletes from labor workers through the distinct characteristics present in urine metabolomics (Chen et al., 2016). This approach offers intriguing insights for our consideration. The variations in urine metabolomic characteristics can be utilized to identify swimmers across different levels of training and competitive status. This is substantiated by the disparities in urine metabolomic features observed amongst swimmers with varying competitive standings (Li et al., 2006). Comparable findings have been corroborated in research focusing on blood metabolomics (Cai et al., 2022). Based on these findings, we attempted to detect the urine metabolites of swimmers at different levels through metabolomics and select different metabolites to establish the model related to their athletic performance and competitive status. Simultaneously, incorporating findings from our preceding investigations on the blood metabolomics of swimmers (Cai et al., 2022), we propose that a multi-component identification model is anticipated to demonstrate enhanced recognition accuracy. This is owing to its assimilation of a more exhaustive range of information, thereby providing a more comprehensive portrayal of the swimmer’s athletic performance and competitive status as compared to a model based on a single component. This study will offer valuable insights and exploratory efforts for refining the practical application of evaluating the athletic status and competitive potential of Chinese swimmers.

## 2 Materials and methods

### 2.1 Ethics approval

This study was conducted in compliance with the Declaration of Helsinki and approved by the Ethics Committee at the School of Life Sciences, Fudan University, China. Written informed consent was obtained from all participants.

### 2.2 Study design

**FIGURE 1:**
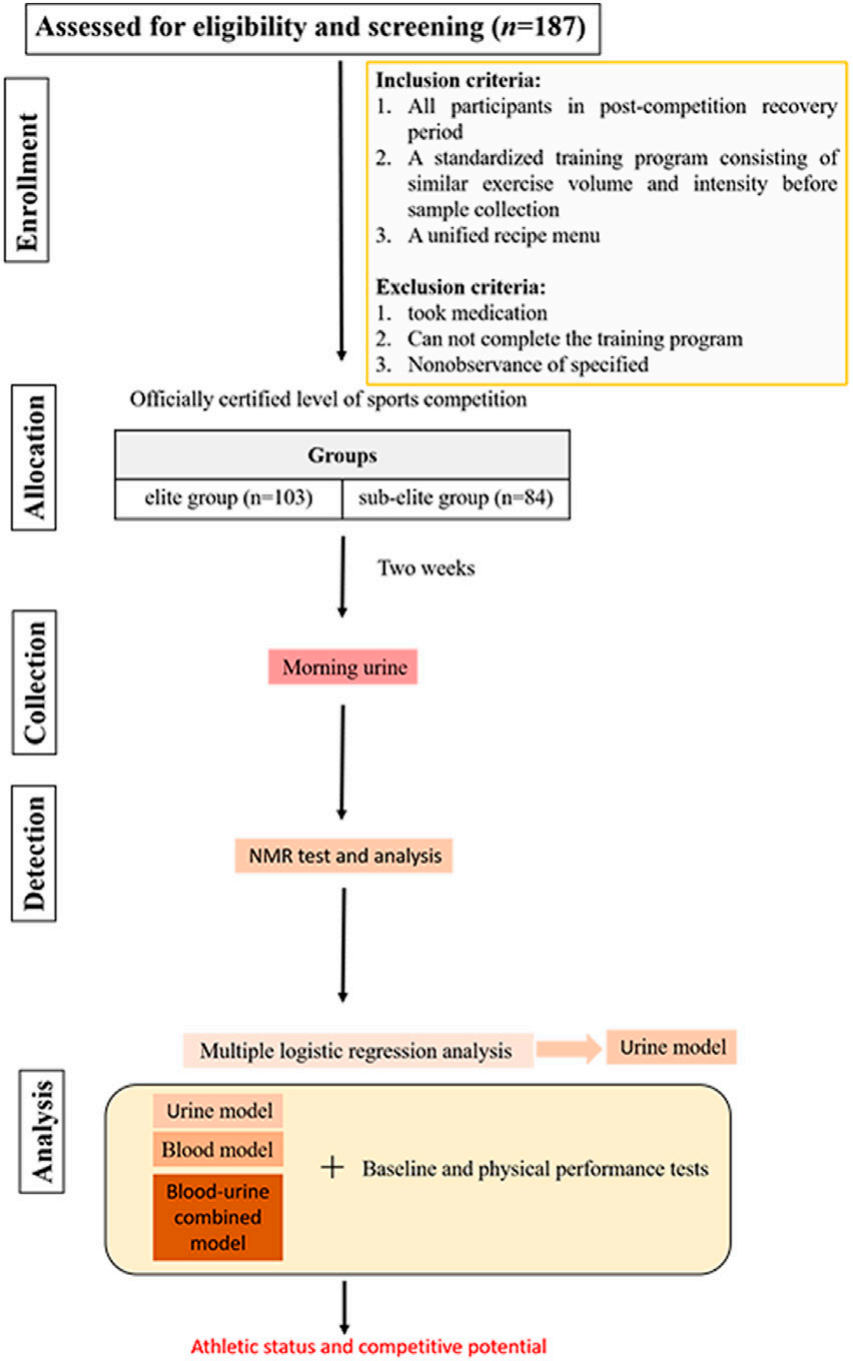
Flowchart of study design.

All participants were in their post-competition recovery period. Two weeks prior to urine sample collection, all swimmers adhered to a standardized training program consisting of similar exercise volume and intensity. The program included 30 min of land exercises before swimming, which consisted of 15 min of stretching exercises and 15 min of relaxation exercises, followed by an 80–90-min swimming session, during which the swimmers completed 4,000 m of swimming at around 60% of their maximum intensity. The session ended with 15 min of relaxation exercises. The daily diet was a unified recipe menu for athletes, which was supervised by the coach in charge and followed from Monday to Sunday at the training base. Athletes who took medication, did not follow the training program, or did not adhere to the diet during the 2 weeks prior to sample collection were excluded from the study. All qualified swimmers were divided into elite and sub-elite groups according to the athletic level certified by the Chinese Swimming Association (the athletic level is determined based on specific competition performance). The elite group consisted of swimmers with a national certification level of International Master and National Master, while the sub-elite group consisted of swimmers with a national certification level of first and second class. After 2 weeks, morning urine samples of all swimmers were collected at the same time point. Prior to the subsequent collection of morning urine, participants were instructed to refrain from eating and urinating upon waking. This ensured that the sampled urine was the first void of the day, post-awakening. All urine samples were analyzed for metabolomics using nuclear magnetic resonance (NMR) spectroscopy. The urine metabolites identified were compared between groups, and multiple logistic regression analysis was utilized to select significant metabolites for modeling. Subsequently, Using the selected urine metabolites, combined with the baseline and physical performance test data, the model was fitted and corrected to find a better model to identify and predict the athletic status and competitive potential of swimmer. By utilizing the data and outcomes of previous study on blood metabolomics (Cai et al., 2022), a comparative analysis was conducted to evaluate the effects of single-component models (either urine or blood metabolites) and combined-component model (urine and blood metabolites) corrected by baseline and physical performance data in identifying and predicting the athletic status and competitive potential of swimmers. The study design process is depicted in Figure 1.

### 2.3 Participants

A total of 103 elite swimmers, comprising 53 male athletes (height = 184.7 ± 5.2 cm, body mass = 78.7 ± 9.3 kg, age range: 18–29 years, training years: >10 years) and 50 female athletes (height = 171.8 ± 5.0 cm, body mass = 62.2 ± 6.3 kg, age range: 16–27 years, training years: >8 years), were recruited from the Shanghai and Zhejiang professional swimming teams who had previously competed at international or national swimming competitions.

The sub-elite group consisted of 84 first- and second-grade swimmers from the Shanghai professional swimming team, Shanghai University of Sport, Shanghai Jiao Tong University, and Tong Ji University. These athletes had participated in provisional or university swimming competitions. Among them, 52 were male (height = 180.1 ± 6.3 cm, body mass = 77.1 ± 10.1 kg, age ranges:17–23 years, training years: >9 years) and 32 were female (height = 168.7 ± 4.9 cm, body mass = 59.8 ± 8.6 kg, age ranges:16–22 years, training years: >8 years).

### 2.4 Urine samples collection and metabolomics analysis

On the second morning after an overnight fast, a 500 μL urine sample was collected from swimmers for metabolomics analysis. To prepare the samples, 14 μL of KF solution (5M) was added to a 1.5 mL EP tube, followed by the addition of 500 μL of urine sample. The mixture was vortexed and left to stand for 10 min, then centrifuged at 4°C and 12,000 rpm for 10 min using a centrifuge 5702R (Eppendorf AG, Hamburg, Germany). For NMR analysis, 20 μL of 0.1M EDTA-d12 was added to a 5 mm NMR tube, and 450 μL of the supernatant from the above centrifugation was added to the NMR tube. Finally, 45 μL of Na+/K+ buffer (1.5M) was added and manually mixed to complete the preparation. All 1D ^1^H NMR spectra of the samples were collected on a Bruker AVIII 600 MHz NMR spectrometer equipped with a super low temperature probe, with the proton resonance frequency set to 600.13 MHz and the experimental temperature set to 298 K. For the analysis of urine samples, the NOESYGPPR1D sequence (RD-90°-t1-90°-tm-90°-ACQ) was used, where RD = 2s, t1 = 4 μs, tm = 100 ms. The spectral width was set to 20ppm, the sampling time was 1.36s, and 32K data points were collected.

Before Fourier transformation, all NMR spectra FID signals were added with an exponential window function with a broadening factor of 1Hz using MestReNova software (MestReNova 8.1, Spain). The TSP (δ 0.00) signal was used for chemical shift calibration, and manual phase and baseline correction were performed. The urine NMR spectra region (0.5–9.5) was integrated using AMIX package (v3.9.2, Bruker Biospin). During the integration process, residual water peak signals and urea signals were removed, and each integration interval size was set to 0.002 ppm. After correcting signals with obvious drift, the total area was normalized. The normalized data was imported into the SIMCA-P+ software package (V.13.0, Umetrics, Sweden) for multivariate statistical analysis, including principal component analysis (PCA) and orthogonal partial least squares discriminant analysis (OPLS-DA). The validity of these assignments was subsequently verified using publicly accessible databases, namely, the Human Metabolome Database (HMDB) and the Biological Magnetic Resonance Bank (BMRB) (Jiang et al., 2012; Song et al., 2015).

PCA was conducted on the data using Centered (Ctr) standardization. The resulting scores were plotted in score plots, which were used primarily to visualize the overall clustering of the samples and to identify any potential outliers. On the other hand, OPLS-DA was conducted on the data using Unit Variance scaling. The results were visualized using score plots and correlation coefficient loading plots. The primary purpose of these plots was to uncover the relationship between the NMR data (X variables) and the grouping information (Y variables). To validate the OPLS-DA model, a 7-fold cross-validation and CV-ANOVA were performed. The CV-ANOVA test yielded a *p*-value < 0.05, indicating a statistically significant model. The standardized data was used for multivariate analysis. The 7-fold cross-validation was performed using soft independent class analogy (SIMCA)-P1 (van 12.0, Umetrics, Sweden). The model was assessed based on R^2^Y, an indicator of the Y variable explained by the model, and Q^2^, which reflects the predictability of the model. To facilitate the biological interpretation of the loads generated in the model, the loads were first inverted and then color-coded using OPLS-DA. The color-coding was based on the absolute value of the OPLS-DA correlation coefficient, |r|. This coefficient represents the contribution of the corresponding variable to the component separation. The significance of the model was further verified using CV-ANOVA with a *p*-value < 0.05.

### 2.5 Assessment of covariates

Participants’ gender, birth date, and years of professional training were obtained through a questionnaire. Body mass index (BMI) was calculated as weight in kilograms divided by height in meters squared. Body fat percentage was assessed using Inbody720 (InBody Co., Ltd., Seoul, South Korea). Standardized test methods (Yang and Shen, 2019) were employed to measure physical performance and function covariates that were relevant to swimmers’ physical performance, such as grip, back strength, standing long jump (SLJ), standing vertical jump (SVJ), abdominal curl, vital capacity, sit-and-reach, acoustic reaction time, and heart rate at rest. Health covariates including hemoglobin, erythropoietin (EPO) and myoglobin (MYO) were tested using an automatic biochemical instrument (Access2, Beckman Inc., United States). 1mL blood sample was collected with 2 mL of EDTA anticoagulant tube for hemoglobin testing, and 2 mL blood sample was collected with 5 mL of heparin lithium anticoagulant tube for EPO and MYO.

### 2.6 Statistical analysis

Baseline characteristics and physical performance measures of elite and sub-elite swimmers were compared using continuous and categorical variables. Continuous variables were expressed as mean ± standard deviation or median (with interquartile range, IQR), whereas categorical variables were depicted as frequencies (percentages), as appropriate. Student’s t-test or Mann-Whitney *U* test was employed to compare continuous variables, while Pearson’s chi-squared test was used for comparisons involving categorical variables. Additionally, the Student’s t-test was applied to compare urine metabolites between elite and sub-elite swimmers and to identify significantly different metabolites. Logistic regression was performed for dimensionality reduction to select metabolomic markers, and correlation analysis was conducted to explore the association between significant metabolites and baseline characteristics. Based on the chosen metabolites and various covariates, three models were established using multivariable logistic regression: Model 1 was unadjusted by any covariate, Model 2 was adjusted by baseline covariates, and Model 3 was adjusted by both baseline and physical performance covariates. To avoid biased estimation, average values of AUC were obtained from 10-fold cross-validation in ROC analysis. The dataset was randomly partitioned into ten subsets, utilizing nine of them sequentially as the training set and one as the test set. The optimal combination of specificity and sensitivity was ascertained by the Youden index method (Youden, 1950). Using data from previous studies and results from blood metabolomics (Cai et al., 2022), ROC analysis was performed to compare the identification and prediction capabilities of the urine metabolites model, serum metabolites model, and combined urine and serum metabolites models. All the analyses were executed using IBM SPSS Statistics 26.0 for Windows and R Studio (R core 4.1.0). A *p*-value of less than 0.05 was considered indicative of statistical significance.

## 3 Results

### 3.1 Baseline characteristics and covariates of swimmers

Table 1 presented a summary of the baseline characteristics, physical performance, and health indicators of all participants according to their athletic status. This study revealed that sub-elite level swimmers exhibited a longer duration of training than their elite counterparts (*p* < 0.001). In terms of physical performance covariates, elite level swimmers demonstrated superior performance in abdominal curl/min and sit-and-reach tests (*p* < 0.001), while displaying lower values in BMI, body fat percentage, vital capacity, and resting heart rate (with *p* < 0.05, *p* < 0.01, and *p* < 0.001, respectively) relative to their sub-elite counterparts. However, no significant differences were observed in other baseline characteristics, physical performance, and health covariates between the two groups (*p* > 0.05).

**TABLE 1:** Baseline, physical performance, and health characteristics of swimmers.

Characteristics	All subjects	Elite level swimmers	Sub-elite level swimmers	*p* value
Participants,n(%)	187(100.0)	103(55.1)	84(44.9)	—
Gender				
Male,n(%)	105(100)	53(50.5)	52(49.5)	0.152
Female,n(%)	82(100)	50(61.0)	32(39.0)	
Age,years, mean ± SD	19.3 ± 2.7	19.0 ± 3.3	19.5 ± 1.7	0.167
Years of professional training, median(IQR)	7.3(4.7,11.5)	6.2(3.9,7.7)	11.1(7.3,13.1)	**<0.001**
BMI,kg/m^2^,mean ± SD	22.5 ± 2.6	22.1 ± 2.2	22.9 ± 2.9	**0.027**
Body fat percentage, %, mean ± SD	16.8 ± 6.5	15.4 ± 5.9	18.6 ± 6.7	**0.001**
Grip,kg,mean ± SD	39.1 ± 10.1	39.3 ± 10.4	38.9 ± 9.8	0.761
Back strength,kg,mean ± SD	100.6 ± 28.8	103.3 ± 28.9	97.3 ± 28.4	0.167
SLJ,cm,mean ± SD	224.7 ± 31.2	228.6 ± 31.7	220.0 ± 30.1	0.064
SVJ,cm,mean ± SD	38.7 ± 8.4	39.2 ± 8.8	38.2 ± 7.9	0.458
Abdominal curl,n/min, mean ± SD	55.2 ± 8.6	57.7 ± 8.2	52.1 ± 8.1	**<0.001**
Vital capacity, litre, mean ± SD	5.1 ± 1.2	4.8 ± 1.1	5.4 ± 1.1	**<0.001**
Sit-and-reach,cm,mean ± SD	20.0 ± 8.3	22.0 ± 8.1	17.6 ± 7.9	**<0.001**
Acoustic reaction time,ms,mean ± SD	260.6 ± 28.1	258.0 ± 26.8	263.7 ± 29.5	0.169
Resting heart rate,n/min,mean ± SD	73.1 ± 11.2	71.1 ± 11.0	75.8 ± 10.9	**0.005**
Hemoglobin,g/L,mean ± SD	141.4 ± 14.7	139.8 ± 14.9	143.4 ± 14.3	0.095
EPO,mIU/mL,mean ± SD	8.6 ± 3.4	8.4 ± 2.5	8.8 ± 4.2	0.446
MYO,ng/mL,median(IQR)	19.3(15.8,23.4)	18.9(15.7,22.5)	19.8(16.0,24.3)	0.141

Abbreviation: BMI, body mass index; SLJ, standing long jump; SVJ, standing vertical jump; EPO, erythropoietin; MYO, myoglobin; SD, standard deviation; IQR, interquartile range. *p* < 0.05 marked in bold.

### 3.2 NMR spectral analysis and urine significant metabolites detection

The ^1^H NMR spectra of urine samples were acquired from all swimmers (Supplementary Figure S1). Identification of specific metabolites was accomplished by assigning resonance peaks to known metabolites through a combination of published data and 2D NMR spectra. Such an approach enhances the reliability and accuracy of the metabolite identification process. Thirty-nine metabolites were identified and assigned, implicating the involvement of multiple interconnected metabolic pathways (Supplementary Table S1).

**FIGURE 2:**
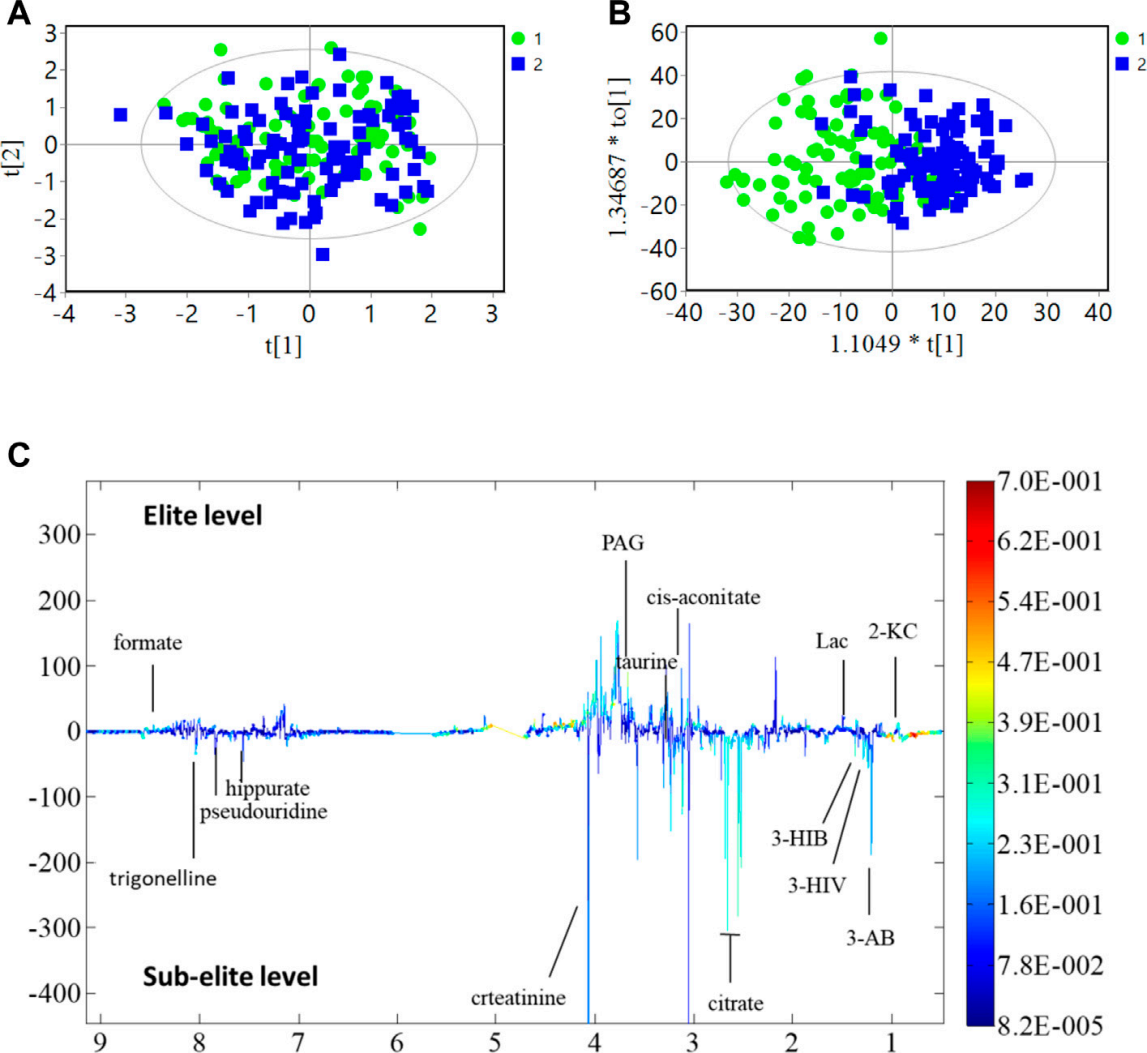
PCA, OPLS-DA analysis and coefficient of urine metabolites. **(A)** PCA analysis; **(B)** OPLS-DA score plot; *R* ^2^ *Y* = 0.507, *Q* ^2^ = 0.167 (*p* < 0.001); **(C)** Multicolor loading graph of urine metabolites analysis. 1: sub-elite level swimmers, 2: elite level swimmers.

The present study investigated differences in urine metabolite profiles between elite and sub-elite level swimmers, utilizing SIMCA statistical methods of PCA (Figure 2A) and OPLS-DA analyses (*R* ^2^ *Y* = 0.507, *Q* ^2^ = 0.167, *p* < 0.001) (Figure 2B). The coefficient plot revealed that 14 metabolites exhibited differential patterns between the two groups, including 2-ketoisocaproate (2-KC), 3-aminobutyrate (3-AB), 3-hydroxyisovalerate (3-HIV), citrate, cis-aconitate, taurine, phenylacetylglycine (PAG), creatinine, hippurate, formate, 3-hydroxyisobutyrate (3-HIB), lactate (Lac), pseudouridine, and trigonelline (Figure 2C).

### 3.3 Metabolites screening and establishment of identification and prediction model

**FIGURE 3:**
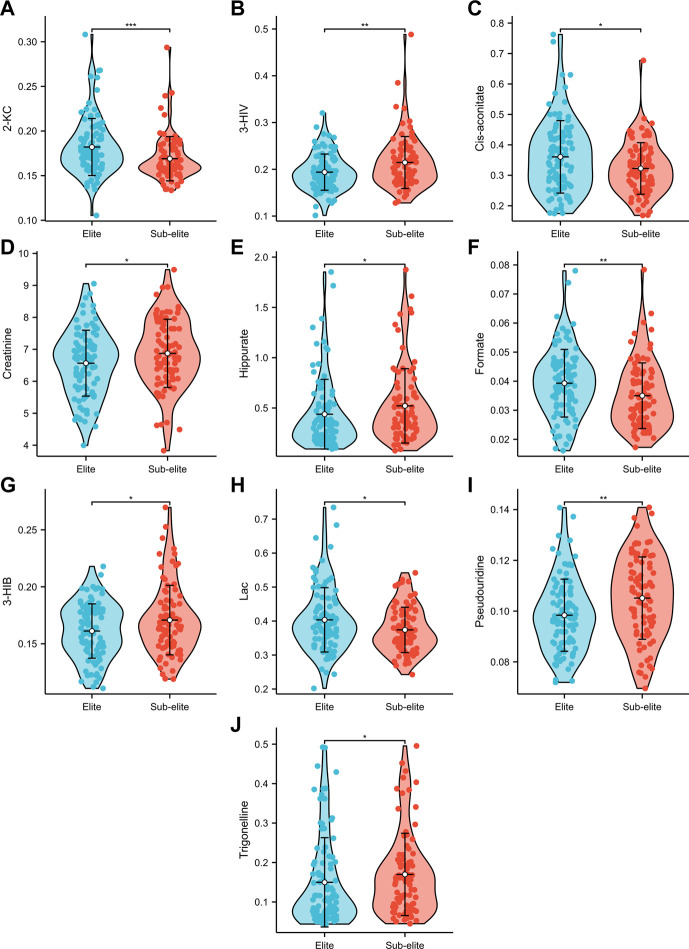
The violin plots of urine metabolites with significant differences between elite and sub-elite level swimmers. **(A)** Comparison of 2-KC between Elite and Sub-elite swimmers. **(B)** Comparison of 3-HIV between Elite and Sub-elite swimmers. **(C)** Comparison of cis-aconitate between Elite and Sub-elite swimmers. **(D)** Comparison of creatinine between Elite and Sub-elite swimmers. **(E)** Comparison of hippurate between Elite and Sub-elite swimmers. **(F)** Comparison of formate between Elite and Sub-elite swimmers. **(G)** Comparison of 3-HIB between Elite and Sub-elite swimmers. **(H)** Comparison of Lac between Elite and Sub-elite swimmers. **(I)** Comparison of pseudouridine between Elite and Sub-elite swimmers. **(J)** Comparison of trigonelline between Elite and Sub-elite swimmers.

Among the 14 metabolites analysed, 10 exhibited differential expression patterns in swimmers categorized as either elite or sub-elite including 2-KC, 3-HIV, cis-aconitiate, creatinine, hippurate, formate, 3-HIB, Lac, pseudouridine, and trigonelline (Table 2). According to our analysis, elite swimmers exhibit significantly higher levels of 2-ketocaproic acid (2-KC), cis-aconitate, formate, and lactate (LAC) in comparison to their sub-elite counterparts. Conversely, levels of 3-hydroxyisovaleric acid (3-HIV), creatinine, 3-hydroxyisobutyric acid (3-HIB), hippurate, pseudouridine, and trigonelline were found to be significantly lower (*p* < 0.05) in elite swimmers (Figure 3). These findings suggest that metabolic profiles may differ significantly between elite and sub-elite swimmers, with elite swimmers exhibiting a distinct metabolic profile.

**TABLE 2:** Urine metabolites with significant differences between elite and sub-elite level swimmers.

	Elite level swimmers vs. sub-elite level swimmers		
	Fold change	*p*-value	adj.*p*-value
2-KC	1.080	**2.31E-04**	**3.24E-03**
3-AB	0.871	2.78E-01	2.99E-01
3-HIV	0.899	**9.13E-03**	**3.20E-02**
citrate	0.848	9.56E-02	1.22E-01
cis-aconitate	1.130	**1.98E-02**	**4.63E-02**
taurine	1.059	1.24E-01	1.45E-01
PAG	1.019	3.91E-01	3.91E-01
creatinine	0.951	**3.52E-02**	**4.93E-02**
hippurate	0.829	**2.71E-02**	**4.93E-02**
formate	1.149	**3.46E-03**	**1.61E-02**
3-HIB	0.941	**1.55E-02**	**4.33E-02**
Lac	1.080	**3.46E-02**	**4.93E-02**
pseudouridine	0.933	**2.75E-03**	**1.61E-02**
trigonelline	0.873	**2.86E-02**	**4.93E-02**

*p* < 0.05 marked in bold. adj.*p*-value: *p*-value after FDR (false discovery rate) correction by the Benjamini Hochberg method.

Multivariable logistic regression was performed on ten metabolites to establish models for identification and prediction of elite and sub-elite swimmers. Among them, 2-KC and 3-HIB were the most significant predictors for the outcome variable of elite or sub-elite swimmers. The associations between these two metabolites and baseline characteristics were examined. Only a few baseline features showed significant but weak correlations with the two metabolites (Table 3).

**TABLE 3:** Correlation analysis between two significant urine metabolites and baseline characteristics.

Characteristics	2-ketoisocaproate		3-hydroxyisobutyrate	
	*r*	*p*-value	*r*	*p*-value
Gender	−0.067	0.375^a^	−0.069	0.362^a^
Age	−0.101	0.185^b^	0.072	0.341^b^
Years of professional training	−0.026	0.728^c^	−0.062	0.417^c^
BMI	−0.052	0.493^d^	0.169	**0.026** ^d^
Body fat percentage	−0.126	0.097^d^	0.067	0.377^d^
Grip	0.048	0.526^d^	0.113	0.137^d^
Back strength	0.065	0.395^d^	0.149	**0.050** ^d^
SLJ	0.088	0.248^d^	−0.008	0.917^d^
SVJ	0.045	0.552^d^	−0.016	0.830^d^
Abdominal curl	0.088	0.247^d^	−0.004	0.953^d^
Vital capacity	−0.019	0.801^d^	0.094	0.216^d^
Sit-and-reach	0.044	0.562^d^	−0.013	0.867^d^
Acoustic reaction time	0.082	0.283^d^	0.043	0.576^d^
Resting heart rate	0.013	0.866^d^	0.060	0.430^d^
Hemoglobin	0.167	**0.028** ^d^	0.159	**0.036** ^d^
EPO	0.061	0.427^d^	0.059	0.436^d^
MYO	−0.090	0.237^d^	0.021	0.786^d^
BUN	0.102	0.182^d^	−0.098	0.197^d^

*r*: correlation coefficient; a: correlation analysis adjusted with sports level (elite and sub-elite), age and years of professional training; b: correlation analysis adjusted with sports level, gender, and years of professional training; c: correlation analysis adjusted with sports level, gender, and age; d: correlation analysis adjusted with sports level, gender, age, and years of professional training. *p* < 0.05 marked in bold.

Three models were generated, including two metabolites unadjusted or adjusted for different covariates. Model one incorporated two metabolites without any covariates, while model two was adjusted for baseline characteristics, including age and years of professional training, using model one as a foundation. Model three, which was built upon model two, was further adjusted for physical performance indicators such as vital capacity and sit-and-reach. In these models, the two urine metabolites were identified as independent influencing factors on the athletic status of swimmers (*p* < 0.05) (Table 4).

**TABLE 4:** Multivariable logistic regression analysis between significant urine metabolites and athletic status in swimmers.

Metabolites	Elite athletic status			AUC
	OR	95%CI	*p-*value	
2-ketoisocaproate				
Model 1	9.87E+09	1.57E+04-6.21E+15	**7.29E-04**	0.709
Model 2	6.13E+08	4.29E+02-8.75E+14	**5.14E-03**	0.828
Model 3	1.79E+07	1.65E+01-1.96E+13	**1.85E-02**	0.852
3-hydroxyisobutyrate				
Model 1	4.50E-09	2.49E-14-8.12E-04	**1.86E-03**	—
Model 2	3.11E-10	2.64E-16-3.67E-04	**2.15E-03**	—
Model 3	2.27E-08	7.71E-15-6.69E-02	**2.06E-02**	—

Model 1 was unadjusted for any covariate; Model 2 was adjusted for age and years of professional training; Model 3 was adjusted for age, years of professional training, vital capacity, and sit-and-reach. *p* < 0.05 marked in bold.

### 3.4 Comparison of three models and selection of the optimal model

**FIGURE 4:**
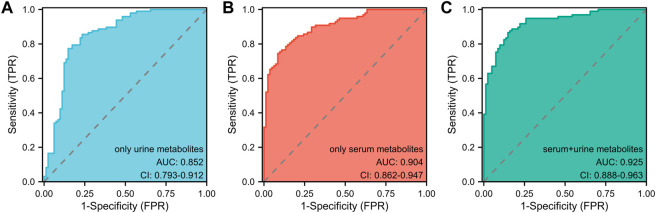
The ROC analyses of three pattern prediction models **(A)** ROC curve analysis of the model only including urine metabolites. AUC: Area Under the Curve. **(B)** ROC curve analysis of the model only including serum metabolites. AUC: Area Under the Curve. **(C)** ROC curve analysis of the model including serum and urine metabolites. AUC: Area Under the Curve.

In light of prior blood metabolism research (Cai et al., 2022), this study incorporated two significant urine metabolites alongside four serum metabolites, establishing a model that combined both blood and urine metabolites with covariates. The effectiveness of three models in determining the athletic status and competitive potential of swimmers was compared using Receiver Operating Characteristic (ROC) analysis (Figure 4): the urine metabolite model (including two urine metabolites), the blood metabolite model (including four blood metabolites), and the blood-urine metabolite combined model (including four blood and two urine metabolites). The Area Under the Curve (AUC) for the urine metabolite model was found to be 0.852 (95% CI: 0.793–0.912) (Figure 4A), while the AUC for the blood metabolite model was 0.904 (95% CI: 0.862–0.947) (Figure 4B) (Cai et al., 2022). The AUC for the blood-urine metabolite combined model was 0.925 (95% CI: 0.888–0.963) (Figure 4C). Among the three models, the blood-urine metabolite combined model demonstrated the highest AUC value and the most effective predicting capability, with an AUC exceeding 0.9. This indicates that the combined model has substantial practical application value within the field.

## 4 Discussion

The combination of both blood and urine samples can provide a more robust predictor of athletes’ exercise level or physical function. In this study, urine metabolites were examined using a high-throughput ^1^H-NMR method, which identified 10 significantly different metabolites between elite and sub-elite swimmers. Among elite swimmers, four metabolites were observed to be higher, while the remaining six were lower. The two most significant metabolites, 2-KC and 3-HIB, were identified through multivariate logistic regression analysis.

In the present study, 2-Ketoisocaproate (2-KC/KIC) levels in elite athletes were higher compared to those in sub-elite athletes. 2-KC is known to reduce the opening of potassium channels and depolarize pancreatic β cells, subsequently leading to increased insulin secretion (Ashcroft et al., 1987). Insulin plays a pivotal role in glycogen formation, protein biosynthesis, and the inhibition of protein breakdown, which may enhance athlete performance and muscle strength (Thevis et al., 2010). KIC serves as the final intermediate in the biosynthesis of l-leucine (Vogt et al., 2015), a branched-chain amino acid (BCAA) that has several critical functions. BCAA can not only augment protein synthesis by activating the mechanistic target of rapamycin (mTOR) signaling pathway in skeletal muscle and adipose tissue, but also boost energy metabolism through glucose uptake, mitochondrial biogenesis, and fatty acid oxidation. Furthermore, it can provide energy for protein synthesis and inhibit protein degradation (Duan et al., 2016). A mouse study demonstrated that exercise activates the mTOR pathway, resulting in neuronal activation and axonal myelination, which in turn improves motor learning (Chen et al., 2019). After aerobic and strength exercises, plasma or serum leucine levels have been found to decrease (Mero, 1999). The result of this study suggests that their leucine levels either decreased to a lesser extent or recovered more rapidly within the body. Consequently, the metabolite 2-KC level may serve as a distinguishing indicator of athletes’ competitive ability and physical fitness.

3-Hydroxyisobutyric acid (3-HIB) is an intermediate in the degradation of valine, a branched-chain amino acid (BCAA) (Letto et al., 1986). 3-HIB, produced by valine, facilitates the uptake of fatty acids in skeletal muscle, resulting in the accumulation of incompletely oxidized lipids in skeletal muscle, which may contribute to insulin resistance in these tissues (Shou et al., 2019). In a mouse study, 3-HIB was secreted from muscle cells, activated endothelial fatty acid transport, stimulated muscle fatty acid uptake, and promoted muscle lipid accumulation, ultimately leading to insulin resistance in mice (Jang et al., 2016). These findings underscore the importance of 3-HIB levels in the body, particularly for athletes. In a metabolomic analysis of marathon runners, valine levels were found to decrease in their serum post-marathon, suggesting that exercise can reduce lipid levels in the body (Shi et al., 2020). Interestingly, BCAAs are a common nutritional supplement for athletes and do not induce insulin resistance. This phenomenon may be attributed to exercise enhancing the mitochondrial oxidation potential of BCAAs, mitigating or even eliminating the accumulation of catabolic intermediates from BCAAs, promoting their catabolism to β-aminoisobutyric acid, increasing plasma β-aminoisobutyric acid concentration, and ultimately improving insulin resistance (Shou et al., 2019). Blood metabolomics studies have shown that elite athletes have lower levels of glutamine. Glutamine can be synthesized from glutamic acid, valine, and isoleucine (Cai et al., 2022), which is consistent with the observed lower levels of 3-HIB in elite athletes in this study. Intriguingly, 2-KC is an intermediate for the synthesis of leucine and 3-HIB is a metabolite of valine, and together with isoleucine, they form BCAAs. This observation suggests that BCAAs play a crucial role in athletes. BCAAs can reduce creatine kinase levels and muscle soreness after vigorous exercise (Fedewa et al., 2019; Doma et al., 2021), and can also enhance innate and adaptive immune responses (Zhang et al., 2017). In research on combat athletes, BCAAs can maintain exercise performance and improve immunity (Trushina et al., 2019). Therefore, there is more BCAA in elite swimmer, aligning with the perspective of blood metabolomics.

Two of the most significant metabolites, 2-KC and 3-HIB, were identified through logistic regression, and a predictive model was established. This study demonstrates that the dual-metabolite model, adjusted for baseline characteristics and physical performance indicators, can effectively discern the athletic performance state of swimmers. For diagnostic or predictive methods, an area under the curve (AUC) value above 0.8 is generally considered meaningful. When comparing the performance of two or more diagnostic tests, the one with the highest AUC value is considered to have better diagnostic or predictive capabilities (Nahm, 2022). In a nuclear magnetic resonance analysis of urine metabolomics, detecting changes in metabolites such as triglycerides, hippurate, and phenylalanine in patients with type 2 diabetes mellitus (DM2) can predict their risk of developing chronic kidney disease (CKD). The best model in this case has an AUC value of 0.912 (Lucio-Gutiérrez et al., 2022). Moreover, L-tyrosine in urinary aromatic amino acid metabolites plays a crucial role in the emergence of agitation (EA) symptoms in pediatric patients during the recovery from general anesthesia. This biomarker has a highly predictive AUC value of 0.81 (Li et al., 2022). In the present study, the best urine-based model has an AUC value of 0.852, which is close to 0.9. This model effectively predicts athletes’ competitive levels and physical quality, providing a theoretical basis for athlete selection. Furthermore, we established a combined urine and blood model with an AUC value of 0.925, which is higher than the AUC values for urine and blood models alone. Consequently, this combined model can more accurately identify and predict athletes’ performance states and physical functions. Similarly, in patients with nasopharyngeal carcinoma, a combined serum and urine metabolomics model demonstrated greater utility, with an AUC value of 0.973, which is higher than the 0.809 AUC value of the urine-only metabolite model. This combined model more effectively distinguishes between healthy individuals and nasopharyngeal carcinoma patients and identifies relevant biomarkers, thus revealing associated metabolic pathways (Liao et al., 2023). Additionally, the combined serum and urine analysis also yields improved results for acute respiratory distress syndrome (Yan et al., 2022). The elevated AUC value associated with the blood-urine combined model signifies enhanced accuracy in differentiating between distinct swim athlete groups and performance levels. This improved predictive capacity facilitates more accurate assessments and informed decision-making for coaches, trainers, and athletes. For instance, ahead of major competitions such as the Olympics, athletes in top competitive status and with adequate preparation can be selected through this model to join the national team. They can represent the country in the competition, aiming to achieve superior performance results. By integrating blood and urine metabolite data, a more comprehensive perspective of an athlete’s physiological state is obtained. This all-encompassing understanding aids in pinpointing factors that impact performance, subsequently guiding targeted interventions for optimal training and recovery. With a precise and thorough comprehension of an athlete’s metabolic profile, sports professionals can devise individualized training programs catering to specific needs, thereby maximizing performance improvements and reducing the likelihood of injury or overtraining. However, there is a dearth of studies that analyze and compare the predictive and recognition capabilities of combined blood and urine models with those of individual blood and urine models in the context of athletic performance. Further research is required to determine whether this method is universally applicable for identifying athletes’ performance states and physical functions across various sports.

Moreover, this study presents several limitations that warrant discussion. Firstly, the sample size was relatively small, which may have a certain impact on the final result. Secondly, the absence of sports interventions for the athletes in this investigation may have resulted in weaker prediction and recognition of their exercise states. In a nuclear magnetic resonance analysis of long-distance runners following 30 min of high-intensity training, significant alterations in lactic acid, glycine, trimethylamine N-oxide (TMAO), and creatinine (CR) levels were observed, which were associated with effective alleviation of post-exercise fatigue based on metabolic product characteristics (Chen and Wang, 2022). In another nuclear magnetic resonance investigation, the metabolic products of football players were assessed one, five, and 10 days post-winter training season (WTS), facilitating the evaluation of athlete recovery and determination of optimal recovery time, thus providing valuable insights for developing training plans. The authors posited that a five-day recovery period following WTS was most appropriate for football players (Kim et al., 2022). Consequently, future research could incorporate swimming and other sports interventions to further examine athletes’ performance and recovery.

In conclusion, the findings of this study demonstrate that urine metabolomics effectively aids in the identification of metabolic biomarkers related to the training status of Chinese professional swimmers. Among the ten distinct urine metabolites discovered, two were more closely associated with the athletes’ exercise status. The metabolic model, established using these two urine metabolites, proficiently identifies and predicts the competitive level and physical condition of swimmers. Utilizing this model and the identified metabolite biomarkers serves as a foundation for differentiating between elite and sub-elite swimmers. Furthermore, the outcomes of urine metabolomics investigations align with previous blood metabolomics studies, providing relevant insights. When comparing the efficacy of urine and blood metabolites, the combined model demonstrates superior utility and guidance for athletes’ sports recognition. Therefore, this study asserts that the combined analysis of urine and blood metabolomics for elite and sub-elite swimmers is more advantageous than urine metabolomics analysis alone.

## Data Availability

The original contributions presented in the study are included in the article/Supplementary Material, further inquiries can be directed to the corresponding author.

## Supplementary material

SUPPLEMENTARY FIGURE S1 Typical urine one-dimensional ^1^H NMR spectra.

SUPPLEMENTARY TABLE S1
^1^H NMR assignments for urine metabolites in swimmers.

## References

[B1] Abdelhafez O. H. Othman E. M. Fahim J. R. Desoukey S. Y. Pimentel-Elardo S. M. Nodwell J. R. (2020). Metabolomics analysis and biological investigation of three Malvaceae plants. Phytochem. Anal. 31, 204–214. 10.1002/pca.2883 31390115

[B2] Ashcroft F. M. Ashcroft S. J. Harrison D. E. (1987). Effects of 2-ketoisocaproate on insulin release and single potassium channel activity in dispersed rat pancreatic beta-cells. J. Physiol. 385, 517–529. 10.1113/jphysiol.1987.sp016505 2443671 PMC1192358

[B3] Bauermeister A. Mannochio-Russo H. Costa-Lotufo L. V. Jarmusch A. K. Dorrestein P. C. (2022). Mass spectrometry-based metabolomics in microbiome investigations. Nat. Rev. Microbiol. 20, 143–160. 10.1038/s41579-021-00621-9 34552265 PMC9578303

[B4] Bongiovanni T. Pintus R. Dessì A. Noto A. Sardo S. Finco G. (2019). Sportomics: Metabolomics applied to sports. The new revolution? *Eur. Rev. Med. Pharmacol.* Sci. 23, 11011–11019. 10.26355/eurrev_201912_19807 31858572

[B5] Brezmes J. Llambrich M. Cumeras R. Gumà J. (2022). Urine NMR metabolomics for precision oncology in colorectal cancer. Int. J. Mol. Sci. 23, 11171. 10.3390/ijms231911171 36232473 PMC9569997

[B6] Cai M. Wu C. Jing C. Shen X. He M. Wang L. (2022). Blood metabolomics analysis identifies differential serum metabolites in elite and sub-elite swimmers. Front. Physiol. 13, 858869. 10.3389/fphys.2022.858869 35600307 PMC9118345

[B7] Castagné R. Boulangé C. L. Karaman I. Campanella G. Santos Ferreira D. L. Kaluarachchi M. R. (2017). Improving visualization and interpretation of metabolome-wide association studies: An application in a population-based cohort using untargeted ^1^H NMR metabolic profiling. J. Proteome. Res. 16, 3623–3633. 10.1021/acs.jproteome.7b00344 28823158 PMC5633829

[B8] Chen K. Zheng Y. Wei J. A. Ouyang H. Huang X. Zhang F. (2019). Exercise training improves motor skill learning via selective activation of mTOR. Sci. Adv. 5, eaaw1888. 10.1126/sciadv.aaw1888 31281888 PMC6609215

[B9] Chen P. Yu Y. B. Huang J. Y. Li H. Y. Dong H. S. Chen B. (2016). Urinary metabonome differentiates athletes and labor workers. Chin. J. Magnetic Reson. 33, 395–405. (In Chinese). 10.11938/cjmr20160304

[B10] Chen Y. Wang X. (2022). Analysis of metabonomic characteristics after exercise fatigue based on NMR. Contrast Media Mol. Imaging 2022, 9041293. 10.1155/2022/9041293 36034195 PMC9392603

[B11] da Cruz J. P. Dos Santos F. N. Rasteiro F. M. Marostegan A. B. Manchado-Gobatto F. B. Gobatto C. A. (2022). A metabolomic approach and traditional physical assessments to compare U22 soccer players according to their competitive level. Biol. (Basel) 11, 1103. 10.3390/biology11081103 PMC9331507 35892959

[B12] Deutsch L. Sotiridis A. Murovec B. Plavec J. Mekjavic I. Debevec T. (2022). Exercise and interorgan communication: Short-term exercise training blunts differences in consecutive daily urine ^1^H-NMR metabolomic signatures between physically active and inactive individuals. Metabolites 12, 473. 10.3390/metabo12060473 35736406 PMC9229485

[B13] Doma K. Singh U. Boullosa D. Connor J. D. (2021). The effect of branched-chain amino acid on muscle damage markers and performance following strenuous exercise: A systematic review and meta-analysis. Appl. Physiol. Nutr. Metab. 46, 1303–1313. 10.1139/apnm-2021-0110 34612716

[B14] Duan Y. Li F. Li Y. Tang Y. Kong X. Feng Z. (2016). The role of leucine and its metabolites in protein and energy metabolism. Amino Acids 48, 41–51. 10.1007/s00726-015-2067-1 26255285

[B15] Fedewa M. V. Spencer S. O. Williams T. D. Becker Z. E. Fuqua C. A. (2019). Effect of branched-chain amino acid supplementation on muscle soreness following exercise: A meta-analysis. Int. J. Vitam. Nutr. Res. 89, 348–356. 10.1024/0300-9831/a000543 30938579

[B16] González-Ravé J. M. Castillo J. A. González-Mohino F. Pyne D. B. (2023). Periodization of altitude training: A collective case study of high-level swimmers. Front. Physiol. 14, 1140077. 10.3389/fphys.2023.1140077 36891142 PMC9986624

[B17] Heaney L. M. Deighton K. Suzuki T. (2019). Non-targeted metabolomics in sport and exercise science. J. Sports Sci. 37, 959–967. 10.1080/02640414.2017.1305122 28346122

[B18] Hsueh C. F. Wu H. J. Tsai T. S. Wu C. L. Chang C. K. (2018). The effect of branched-chain amino acids, citrulline, and arginine on high-intensity interval performance in young swimmers. Nutrients 10, 1979. 10.3390/nu10121979 30558143 PMC6315994

[B19] Jang C. Oh S. F. Wada S. Rowe G. C. Liu L. Chan M. C. (2016). A branched-chain amino acid metabolite drives vascular fatty acid transport and causes insulin resistance. Nat. Med. 22, 421–426. 10.1038/nm.4057 26950361 PMC4949205

[B20] Jiang L. Huang J. Wang Y. Tang H. (2012). Metabonomic analysis reveals the CCl4-induced systems alterations for multiple rat organs. J. Proteome Res. 11, 3848–3859. 10.1021/pr3003529 22612988

[B21] Julian R. Meyer T. Fullagar H. H. Skorski S. Pfeiffer M. Kellmann M. (2017). Individual patterns in blood-borne indicators of fatigue-trait or chance. J. Strength Cond. Res. 31, 608–619. 10.1519/JSC.0000000000001390 28212266

[B22] Khoramipour K. Sandbakk Ø. Keshteli A. H. Gaeini A. A. Wishart D. S. Chamari K. (2022). Metabolomics in exercise and sports: A systematic review. Sports Med. 52, 547–583. 10.1007/s40279-021-01582-y 34716906

[B23] Kim H. Y. Lee J. D. Lee Y. H. Seo S. W. Lee H. S. Kim S. (2022). Urinary metabolomics in young soccer players after winter training season. Metabolites 12, 1283. 10.3390/metabo12121283 36557321 PMC9784126

[B24] Klein D. J. Anthony T. G. McKeever K. H. (2021). Metabolomics in equine sport and exercise. J. Anim. Physiol. Anim. Nutr. Berl. 105, 140–148. 10.1111/jpn.13384 32511844

[B25] Koopmann T. Faber I. Baker J. Schorer J. (2020). Assessing technical skills in talented youth athletes: A systematic review. Sports Med. 50, 1593–1611. 10.1007/s40279-020-01299-4 32495253 PMC7441090

[B26] Lahart I. M. Metsios G. S. (2018). Chronic physiological effects of swim training interventions in non-elite swimmers: A systematic review and meta-analysis. Sports Med. 48, 337–359. 10.1007/s40279-017-0805-0 29086218

[B27] Letto J. Brosnan M. E. Brosnan J. T. (1986). Valine metabolism. Gluconeogenesis from 3-hydroxyisobutyrate. Biochem. J. 240, 909–912. 10.1042/bj2400909 3827880 PMC1147506

[B28] Li J. H. Liu C. Y. Xu X. Y. Ruan C. X. (2006). Metabolic study on sprint swimmer participating in the 15^th^ doha asian games. China Sport Sci. 28, 42–46. 10.16469/j.css.2008.02.007

[B29] Li Y. Li J. Shi Y. Zhou X. Feng W. Han L. (2022). Urinary aromatic amino acid metabolites associated with postoperative emergence agitation in paediatric patients after general anaesthesia: Urine metabolomics study. Front. Pharmacol. 13, 932776. 10.3389/fphar.2022.932776 35928271 PMC9343964

[B30] Liao Z. Zhao L. Zhong F. Zhou Y. Lu T. Liu L. (2023). Serum and urine metabolomics analyses reveal metabolic pathways and biomarkers in relation to nasopharyngeal carcinoma. Rapid Commun. Mass Spectrom. 37, e9469. 10.1002/rcm.9469 36593223

[B31] Lucio-Gutiérrez J. R. Cordero-Pérez P. Farías-Navarro I. C. Tijerina-Marquez R. Sánchez-Martínez C. Ávila-Velázquez J. L. (2022). Using nuclear magnetic resonance urine metabolomics to develop a prediction model of early stages of renal disease in subjects with type 2 diabetes. J. Pharm. Biomed. Anal. 219, 114885. 10.1016/j.jpba.2022.114885 35779355

[B32] Lv Z. Gong Z. G. Xu Y. J. (2022). Research in the field of exercise and metabolomics: A bibliometric and visual analysis. Metabolites 12, 542. 10.3390/metabo12060542 35736475 PMC9230385

[B33] Mero A. (1999). Leucine supplementation and intensive training. Sports Med. 27, 347–358. 10.2165/00007256-199927060-00001 10418071

[B34] Moreira L. P. Silveira L. Jr. Pacheco M. T. T. da Silva A. G. Rocco D. D. F. M. (2018). Detecting urine metabolites related to training performance in swimming athletes by means of Raman spectroscopy and principal component analysis. J. Photochem. Photobiol. B 185, 223–234. 10.1016/j.jphotobiol.2018.06.013 29966989

[B35] Muthubharathi B. C. Gowripriya T. Balamurugan K. (2021). Metabolomics: Small molecules that matter more. Mol. Omics. 17, 210–229. 10.1039/d0mo00176g 33598670

[B36] Nahm F. S. (2022). Receiver operating characteristic curve: Overview and practical use for clinicians. Korean J. Anesthesiol. 75, 25–36. 10.4097/kja.21209 35124947 PMC8831439

[B37] Pal S. Rendedula D. Kumar Nagendla N. Kaliyaperumal M. Krishna Reddy Mudiam M. Mahmood Ansari K. (2022). Serum and urine metabolomics analysis reveals the role of altered metabolites in patulin-induced nephrotoxicity. Food Res. Int. 156, 111177. 10.1016/j.foodres.2022.111177 35651038

[B38] Pla R. Le Meur Y. Aubry A. Toussaint J. F. Hellard P. (2019). Effects of a 6-week period of polarized or threshold training on performance and fatigue in elite swimmers. Int. J. Sports Physiol. Perform. 14, 183–189. 10.1123/ijspp.2018-0179 30040002

[B39] San-Millán I. (2019). Blood biomarkers in sports medicine and performance and the future of metabolomics. Methods Mol. Biol. 1978, 431–446. 10.1007/978-1-4939-9236-2_26 31119678

[B40] Shi R. Zhang J. Fang B. Tian X. Feng Y. Cheng Z. (2020). Runners' metabolomic changes following marathon. Nutr. Metab. (Lond.) 17, 19. 10.1186/s12986-020-00436-0 32190096 PMC7071712

[B41] Shou J. Chen P. J. Xiao W. H. (2019). The effects of BCAAs on insulin resistance in athletes. J. Nutr. Sci. Vitaminol. (Tokyo) 65, 383–389. 10.3177/jnsv.65.383 31666474

[B42] Song Y. Zhao R. Hu Y. Hao F. Li N. Nie G. (2015). Assessment of the biological effects of a multifunctional nano-drug-carrier and its encapsulated drugs. J. Proteome Res. 14, 5193–5201. 10.1021/acs.jproteome.5b00513 26531143

[B43] Talmor-Barkan Y. Bar N. Shaul A. A. Shahaf N. Godneva A. Bussi Y. (2022). Metabolomic and microbiome profiling reveals personalized risk factors for coronary artery disease. Nat. Med. 28, 295–302. 10.1038/s41591-022-01686-6 35177859 PMC12365913

[B44] Tang F. C. (2006). Influence of branched-chain amino acid supplementation on urinary protein metabolite concentrations after swimming. J. Am. Coll. Nutr. 25, 188–194. 10.1080/07315724.2006.10719531 16766776

[B45] Thevis M. Thomas A. Schänzer W. (2010). Insulin. Handb. Exp. Pharmacol. 195, 209–226. 10.1007/978-3-540-79088-4_10 20020367

[B46] Trushina E. N. Vybornov V. D. Riger N. A. Mustafina O. K. Solntsevа T. N. Timonin A. N. (2019). The efficiency of branched chain aminoacids (BCAA) in the nutrition of combat sport athletes. Vopr. Pitan. 88, 48–56. Russian. 10.24411/0042-8833-2019-10041 31722141

[B47] Vogt M. Haas S. Polen T. van Ooyen J. Bott M. (2015). Production of 2-ketoisocaproate with Corynebacterium glutamicum strains devoid of plasmids and heterologous genes. Microb. Biotechnol. 8, 351–360. 10.1111/1751-7915.12237 25488800 PMC4353348

[B48] Wang R. Li B. Lam S. M. Shui G. (2020). Integration of lipidomics and metabolomics for in-depth understanding of cellular mechanism and disease progression. J. Genet. Genomics 47, 69–83. 10.1016/j.jgg.2019.11.009 32178981

[B49] Wang R. Wu X. Lin K. Guo S. Hou Y. Ma R. (2022). Plasma metabolomics reveals β-glucan improves muscle strength and exercise capacity in athletes. Metabolites 12, 988. 10.3390/metabo12100988 36295890 PMC9607031

[B50] Wang X. Niu C. Lu J. Li N. Li J. (2014). Hydrolyzed protein supplementation improves protein content and peroxidation of skeletal muscle by adjusting the plasma amino acid spectrums in rats after exhaustive swimming exercise: A pilot study. J. Int. Soc. Sports Nutr. 11, 5. 10.1186/1550-2783-11-5 24565110 PMC3945952

[B51] Weiss A. Alack K. Klatt S. Zukunft S. Schermuly R. Frech T. (2022). Sustained endurance training leads to metabolomic adaptation. Metabolites 12, 658. 10.3390/metabo12070658 35888781 PMC9323347

[B52] Yan Y. Chen J. Liang Q. Zheng H. Ye Y. Nan W. (2022). Metabolomics profile in acute respiratory distress syndrome by nuclear magnetic resonance spectroscopy in patients with community-acquired pneumonia. Respir. Res. 23, 172. 10.1186/s12931-022-02075-w 35761396 PMC9235271

[B53] Yang R. Shen X. (2019). A phenomics study in Chinese elite swimmers. J. Beijing Sport Univ. 42, 17–22. (In Chinese). 10.19582/j.cnki.11-3785/g8.2019.07.003

[B54] Youden W. J. (1950). Index for rating diagnostic tests. Cancer 3, 32–35. 10.1002/1097-0142(1950)3:1<32:aid-cncr2820030106>3.0.co;2-3 15405679

[B55] Zhang S. Zeng X. Ren M. Mao X. Qiao S. (2017). Novel metabolic and physiological functions of branched chain amino acids: A review. J. Anim. Sci. Biotechnol. 8, 10. 10.1186/s40104-016-0139-z 28127425 PMC5260006

