# Supplementary material for: The combined analysis of urine and blood metabolomics profiles provides an accurate prediction of the training and competitive status of Chinese professional swimmers
Source: Front Physiol. 2023 Jun 14;14:1197224. doi: 10.3389/fphys.2023.1197224 (PMC10307620; doi:10.3389/fphys.2023.1197224)
Supplement: Supplementary file 1 [file Table1.docx]

**Table S1** ^1^H NMR assignments for urine metabolites in swimmers

| NO. | Metabolites | Moieties | δ 1H (ppm) and multiplicity |
| --- | --- | --- | --- |
| 1 | 3-aminobutyrate (3-AB) | CH3 | 1.20(d) |
|  |  | CH | 2.62(m) |
|  |  | CH2 | 3.04(dd) |
|  |  | CH2’ | 3.10(dd) |
| 2 | lactate (Lac) | αCH | 4.12(q) |
|  |  | βCH3 | 1.34(d) |
|  |  | COOH |  |
| 3 | 3-hydroxyisovalerate (3-HIV) | CH3 | 1.28(s) |
|  |  | CH2 | 2.38(s) |
| 4 | alanine (Ala) | αCH | 3.80(q) |
|  |  | βCH3 | 1.49(d) |
|  |  | COOH |  |
| 5 | citrate | CH2 | 2.54(d) |
|  |  | CH2' | 2.67(d) |
|  |  | C-OH |  |
|  |  | COOH |  |
| 6 | dimethylamine (DMA) | CH3 | 2.72(s) |
| 7 | creatinine | CH3 | 3.05(s) |
|  |  | CH2 | 4.06(s) |
|  |  | C=NH |  |
| 8 | creatine (Cr) | CH3 | 3.04(s) |
|  |  | CH2 | 3.94(s) |
|  |  | C=NH |  |
| 9 | trimethylamine N-oxide(TMAO) | CH3 | 3.28(s) |
| 10 | taurine (Tau) | CH2SO3 | 3.29(t) |
|  |  | CH2NH2 | 3.42(t) |
| 11 | scyllo-inosoitol | CH | 3.36(s) |
| 12 | acetamide | CH3 | 1.99(s) |
|  |  | C=O |  |
| 13 | 3-hydroxyisobutyrate (3-HIB) | CH3 | 1.08(d) |
|  |  | CH | 2.5(m) |
|  |  | CH2 | 3.5(dd) |
|  |  | CH2’ | 3.7(dd) |
| 14 | succinate (Succ) | CH2 | 2.41(s) |
|  |  | COOH |  |
| 15 | glycine (Gly) | CH2 | 3.56(s) |
|  |  | COOH |  |
| 16 | α-glucose (α-Glc) | 1CH | 5.26(d) |
|  |  | 2CH | 3.54(dd) |
|  |  | 3CH | 3.73(dd) |
|  |  | 4CH | 3.42(dd) |
|  |  | 5CH | 3.83(dd) |
|  |  | 6CH2 | 3.83(dd) |
| 17 | β–glucose (β-Glc) | 1CH | 4.66(d) |
|  |  | 2CH | 3.25(dd) |
|  |  | 3CH | 3.50(dd) |
|  |  | 4CH | 3.40(dd) |
|  |  | 5CH | 3.47(dd) |
|  |  | 6CH | 3.74(dd) |
|  |  | 6'CH | 3.90(dd) |
| 18 | cis-aconitate | CH | 5.68(s) |
|  |  | CH2 | 3.12(s) |
|  |  | COOH |  |
| 19 | urea | (NH2)2 | 5.79(brs) |
| 20 | p-Cresol sulfate | CH3 | 2.35(s) |
|  |  | 2,6CH | 7.21(d) |
|  |  | 3,5CH | 7.30(d) |
| 21 | 4-hydroxyphenylacetate (4-HPA) | 2,6CH | 6.89(d) |
|  |  | 3,5CH  CH2 | 7.18(m)  3.45(s) |
|  |  | COOH |  |
| 22 | indoxyl sulfate | C9H | 7.71(d) |
|  |  | C6H | 7.51(d) |
|  |  | C2H | 7.36(s) |
|  |  | C7H | 7.28(t) |
|  |  | C8H | 7.21(t) |
| 23 | hippurate | 3,5CH | 7.56(dd) |
|  |  | 4CH | 7.65(t) |
|  |  | 2,6CH | 7.84(dd) |
|  |  | αCH2 | 3.97(d) |
|  |  | COOH |  |
| 24 | 4-hydroxybenzoic acid | CH2 | 3.45(s) |
|  |  | 2,6CH | 6.87(d) |
|  |  | 3,5CH | 7.17(d) |
| 25 | propylene glycol | CH3 | 1.15(d) |
|  |  | CH2 | 3.45(dd) |
|  |  | CH2’ | 3.56(dd) |
|  |  | CH | 2.90(m) |
| 26 | N1-methyl-2-pyridone-3-carboxamide (2PY) | 3CH | 6.67(d) |
|  |  | 4CH | 7.98(d) |
|  |  | 6CH | 8.33(d) |
| 27 | guanidoacetate (GA) | CH2 | 3.80(s) |
|  |  | C=NH |  |
|  |  | COOH |  |
| 28 | formate | CH | 8.46(s) |
|  |  | COOH |  |
| 29 | 2-ketoisocaproate (2-KC) | (CH3)2 | 0.94(d) |
|  |  | CH | 2.00(m) |
|  |  | CH2 | 2.18(d) |
|  |  | O=C-N |  |
| 30 | pseudouridine | CH(ring) | 7.69(s) |
|  |  | 1C'H(ribose) | 4.68(d) |
|  |  | 2C'H(ribose) | 4.31(dd) |
|  |  | 3C'H(ribose) | 4.16(dd) |
|  |  | 4C'H(ribose) | 4.05(dd) |
|  |  | CH2OH | 4.03(dd) |
|  |  | CH2'OH | 4.01(dd) |
| 31 | 3-methyl-2-oxovalerate (3-MOV) | CH3 | 0.88(t) |
|  |  | CH3 | 1.10(d) |
|  |  | CH2a | 1.47(m) |
|  |  | CH2b | 1.70(m) |
|  |  | CH | 2.93(m) |
|  |  | COOH |  |
| 32 | trigonelline | 1CH | 9.13(s) |
|  |  | 2, 4CH | 8.84(dd) |
|  |  | 3CH | 8.04(d) |
|  |  | CH3 | 4.45(s) |
| 33 | 2-hydroxyisobutyrate (2-HIB) | CH3 | 1.36(s) |
|  |  | COOH |  |
| 34 | valerate | δCH3 | 0.88(t) |
|  |  | γCH2 | 1.31(m) |
|  |  | βCH2 | 1.60(m) |
|  |  | αCH2 | 2.28(m) |
|  |  | COOH |  |
| 35 | 1-Methylnicotinamide | CH3 | 4.49(s) |
|  |  | C5H | 8.19(m) |
|  |  | C4H | 8.90(m) |
|  |  | C6H | 8.98(m) |
|  |  | C6H | 9.27(m) |
| 36 | acetate | CH3 | 1.93(s) |
|  |  | COOH |  |
| 37 | phenylacetylglycine (PAG) | 3,5CH | 7.43(m) |
|  |  | 2,6CH | 7.37(m) |
|  |  | 4CH  CH2-NH | 7.37(m)  3.76(d) |
|  |  | CH2 | 3.68(s) |
|  |  | C=O |  |
| 38 | phenylacetylglutamine | C*H*2CONH | 3.65(s) |
|  |  | 2,4,6CH | 7.37(m) |
|  |  | 3,5CH | 7.43(m) |
| 39 | trimethylamine (TMA) | CH3 | 2.88(s) |
| U1 |  |  | 1.24(d) |
| U2 |  |  | 3.11(s) |
| U3 |  |  | 2.83(s) |
| U4 |  |  | 2.95(s) |
| U5 |  |  | 3.76(s) |
| U6 |  |  | 1.66(m) |
|  |  |  | 1.74(m) |
|  |  |  | 1.87(d) |
| U7 |  |  | 3.23(s) |
| s, singlet; d, doublet; t, triplet; q, quartet; m, multiplet; dd, doublet of doublet; brs, broad singlet. | | | |

U1-U7: unknown metabolites
