# Supplementary figures and images for: The combined analysis of urine and blood metabolomics profiles provides an accurate prediction of the training and competitive status of Chinese professional swimmers
Source: Front Physiol. 2023 Jun 14;14:1197224. doi: 10.3389/fphys.2023.1197224 (PMC10307620; doi:10.3389/fphys.2023.1197224)

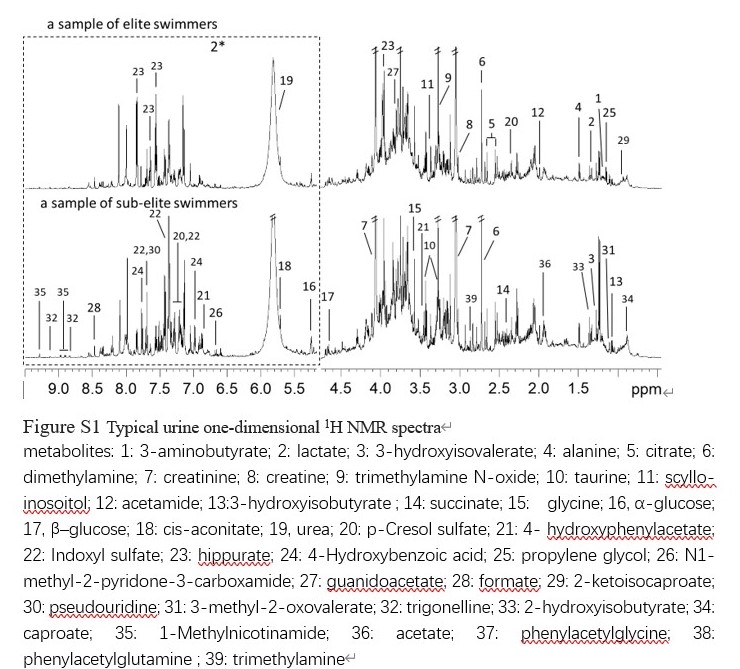

Supplement: Supplementary file 2 [file Image1.jpg]
